# Vaginal Inoculation of Uropathogenic Escherichia coli during Estrus Leads to Genital and Renal Colonization

**DOI:** 10.1128/iai.00532-21

**Published:** 2022-03-31

**Authors:** Christen K. Robinson, Panatda Saenkham-Huntsinger, Braden S. Hanson, L. Garry Adams, Sargurunathan Subashchandrabose

**Affiliations:** a Comparative Medicine Program, College of Veterinary Medicine and Biomedical Sciences, Texas A&M University, College Station, Texas, USA; b Department of Veterinary Pathobiology, College of Veterinary Medicine and Biomedical Sciences, Texas A&M University, College Station, Texas, USA; Yale University School of Medicine

**Keywords:** mouse model of UTI, UPEC, vaginal inoculation, urogenital infection, CFT073, UTI89

## Abstract

Urinary tract infection (UTI) is one of the most prevalent bacterial infections, particularly in women, children, and the elderly. Uropathogenic Escherichia coli (UPEC) is the predominant etiological agent of UTI. Uropathogens are directly instilled in the urinary bladder, bypassing the lower urogenital tract, in the widely used murine model of UTI. We assessed whether vaginal inoculation of UPEC led to UTI and how stages of the estrous cycle would impact bacterial colonization in mice. Mice in proestrus, estrus, metestrus, and diestrus were identified by vaginal cytology and inoculated with UPEC in the vaginal tract. Mice were euthanized 1 day after infection, and bacterial loads in the urogenital tract, liver, and spleen were enumerated. Mice in estrus exhibited the highest and most consistent UPEC burdens in all organs, except the bladder. Vaginal inoculation resulted in bladder colonization in a UPEC strain-specific manner. In contrast, transurethral inoculation of UPEC led to bladder colonization. Importantly, inoculation by both routes led to vaginal and uterine colonization and concomitant systemic dissemination to the spleen and liver. The kinetics of bacterial colonization over 2 weeks following vaginal inoculation was comparable in the urogenital tract. Tissue sections revealed the induction of vaginitis and cystitis upon the vaginal instillation of UPEC. In summary, vaginal inoculation of UPEC in mice during estrus represents a novel approach to investigate infection of the kidneys and genital tract and systemic dissemination from the urogenital tract. Our findings suggest that estrogen primes the urogenital tract to create a conducive milieu for UPEC colonization.

## INTRODUCTION

Bacterial infection of the urinary bladder is a ubiquitous infectious condition (1). Urinary tract infection (UTI) results in ∼11 million physician office visits, 1.7 million emergency room visits, and 470,000 hospitalizations, with an annual direct cost of approximately $3.5 billion in the United States (2–5). Cystitis, inflammation caused by infection of the urinary bladder, is the most common clinical presentation of UTI (6). Less common but more serious outcomes of UTI include kidney infection (pyelonephritis), bacteremia, and sepsis (6, 7). Women are at a 4-times-higher risk of developing UTI than men due to anatomic differences in the urogenital tract (8). Factors that increase the risk for UTI include diabetes mellitus, age (children and the elderly), catheter use, anatomic/physiological abnormalities of the urinary tract, or urolithiasis (7, 9, 10). Recurrent UTI is also common among high-risk groups. Uropathogenic Escherichia coli (UPEC) is the predominant etiological agent of UTI (7, 9, 11). Uropathogens are found in the human gut microbiome (12, 13) and infect the urinary bladder by colonizing the lower urogenital tract followed by ascension along the urethra (14).

Mouse models are used to elucidate the pathogen and host factors that determine the outcome of UTI (15–19). Murine models currently used to investigate experimental UTI rely on the intravesical instillation of uropathogens through a transurethral catheter. Colonization of the urinary bladder with uropathogens results in cystitis, inflammation of the urinary bladder. Uropathogens often cause ascending infections leading to pyelonephritis and systemic dissemination from the urinary tract. UPEC and other uropathogens are known to establish an intracellular niche within the urothelium that lines the bladder lumen (20–23). Recently, UPEC has been demonstrated to invade and form intracellular reservoirs in vaginal epithelial cells, reminiscent of the phenomenon observed in the urothelium (24). Sites of inoculation of uropathogens in the existing murine models of UTI bypass lower urogenital tract colonization to directly introduce uropathogens in the urinary bladder. A better understanding of the mechanisms involved in uropathogen colonization in the lower urogenital tract and their impact on the development of UTI is critical for developing novel intervention strategies against this important public health problem.

Here, we sought to evaluate the impact of vaginal inoculation of UPEC on colonization of the urogenital tract in female CBA/J mice. We also assessed which stage of the estrous cycle would be more permissive to UPEC colonization. Our results reveal that vaginal instillation of UPEC led to consistent genital and renal colonization for up to 2 weeks. However, vaginal instillation results in poor bladder colonization, compared to intravesical instillation of UPEC. Furthermore, a UPEC strain-dependent effect on bladder colonization was also evident after intravaginal inoculation. In summary, we present a novel approach to investigate urogenital colonization following inoculation of UPEC in the vaginal tract.

## RESULTS

### Vaginal instillation of UPEC during various stages of the estrous cycle.

**FIG 1:**
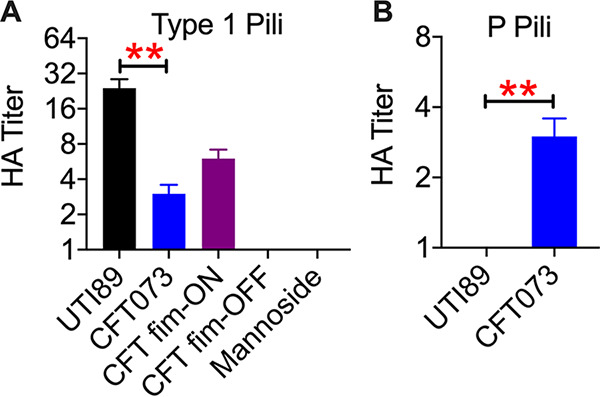
Type 1 and P pilus production in UPEC. Agglutination of guinea pig (A) and human (B) erythrocytes by clinical UPEC strains (UTI89 and CFT073) was evaluated. Mutants in strain CFT073 that constitutively produce type 1 pili (CFT fim-ON) and lack type 1 pili (CFT fim-OFF) were used as positive and negative controls, respectively. Mannoside is an inhibitor of type 1 pilus-mediated hemagglutination (HA) and was used as a negative control. Means and standard errors of the means (SEM) from independent experiments are presented. **, *P < *0.01 (by a *t* test).

**FIG 2:**
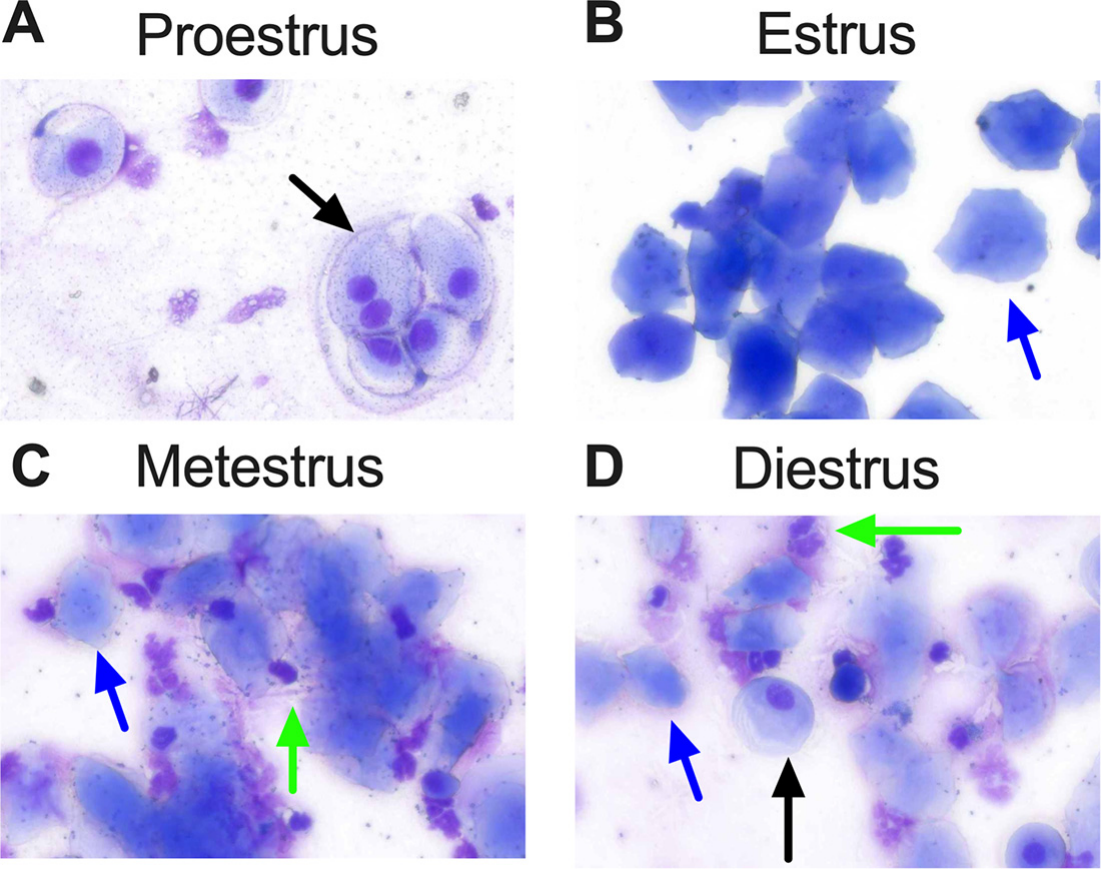
Vaginal cytology reveals estrous cycle-associated changes. Mice were instilled with UPEC strain CFT073 in the vagina during various stages of the estrous cycle. Cytological evaluation of vaginal lavage fluid was performed at 1 day postinoculation. (A) Nucleated epithelial cells (black arrow) were observed during proestrus. (B) Estrus was characterized by the presence of cornified epithelial cells (blue arrow). (C) Cornified and polymorphonuclear cells (green arrow) were evident during metestrus. (D) Diestrus was marked by the presence of all these cell types. A representative image for each stage of the estrous cycle at a ×400 magnification is included here.

First, we assessed the effects of the stage of the estrous cycle on UPEC colonization in the urogenital tract of female CBA/J mice. Vaginal cytology was used to establish that these mice were cycling and to assess the stage of the estrous cycle at the time of bacterial instillation. UPEC strain CFT073 (∼2 × 10^8^ CFU) was instilled into the vaginal tract (*n* = 8 to 14 mice/stage of the estrous cycle). The presence of type 1 and P pili, well-characterized virulence factors of UPEC, was evaluated by agglutination of guinea pig and human erythrocytes, respectively (25, 26). UPEC strain CFT073 produced both type 1 and P pili but with a lower level of type 1 pili than strain UTI89 (Fig. 1A and B). This assay was validated with UPEC mutants and a chemical inhibitor (Fig. 1A). Strain UTI89 produced a higher level of type 1 pili than CFT073 and did not produce type P pili (Fig. 1A and B). Mice were euthanized at 1 day postinoculation, and bacterial loads in the urine, vaginal lavage fluid, blood, and organs (urogenital tract, spleen, and liver) were determined. We did not detect UPEC in blood samples by plate counts (limit of detection = 10 CFU/mL). Cytological evaluation of vaginal lavage fluid revealed the presence of nucleated epithelial cells during proestrus (Fig. 2A), cornified epithelial cells during estrus (Fig. 2B), a mixture of cornified cells and polymorphonuclear cells during metestrus (Fig. 2C), and all these cell types during diestrus (Fig. 2D). A few epithelial cells were detected during urine cytology, but they did not appear to harbor UPEC (see Fig. S1 in the supplemental material).

### Mice in estrus and metestrus exhibit higher bacterial colonization.

**FIG 3:**
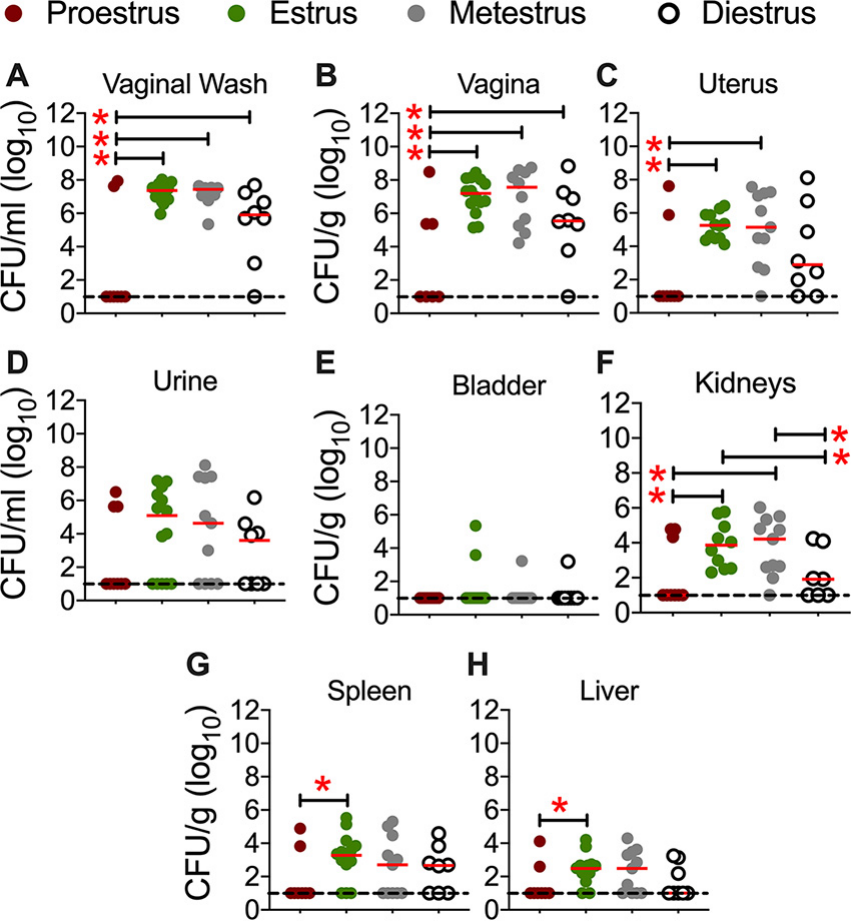
UPEC colonization in the urogenital tract during various stages of the estrous cycle. Female CBA/J mice (*n* = 8 to 14 mice/stage of the estrous cycle) were inoculated with UPEC strain CFT073 in the vaginal tract. Urine and vaginal lavage fluid were collected at 1 day postinoculation, prior to euthanasia. Bacterial loads in the samples were determined by viable counts and normalized to the volume/weight of the samples. Each symbol corresponds to results from a mouse, and red bars indicate medians. Data from two replicates are depicted here. The dotted lines correspond to the limit of detection (10 CFU). *, *P < *0.05 (by a Mann-Whitney test).

Mice in proestrus were poorly colonized by UPEC, with bacterial counts below the limit of detection (10 CFU) in many mice at all sites and samples that were evaluated (Fig. 3A to H). Bacterial colonization was higher in mice in other stages of the estrous cycle at all sites/samples that were evaluated (Fig. 3A to H).

Mice in estrus and metestrus were more susceptible to UPEC colonization in the genital tract than mice in proestrus and diestrus (Fig. 3A to C). Mice in estrus (*n* = 14; 100%) and metestrus (*n* = 11; 91%) had detectible UPEC colonization in the genital tract (Fig. 3A to C). Although the urine bacterial load was highest in mice during estrus relative to other stages, this difference was not statistically significant (Fig. 3D). Instillation of UPEC strain CFT073 in the vaginal tract did not lead to effective colonization of the urinary bladder (Fig. 3E), regardless of the stage of the estrous cycle. The kidney UPEC load was higher in mice in estrus and metestrus than in those in proestrus and diestrus (Fig. 3F). The tissue bacterial loads of mice in estrus and metestrus did not differ significantly from each other (Fig. 3A to H). During diestrus, compared with proestrus, mice had elevated UPEC loads in the vaginal wash fluid and vagina but not in the uterus (Fig. 3A to C).

### UPEC disseminates from the vagina to systemic sites.

Mice in estrus also exhibited more systemic dissemination from the urogenital tract to the spleen and liver than those in proestrus (Fig. 3G and H). While there were increases in spleen and liver UPEC loads during metestrus and diestrus, these differences were not statistically significant. UPEC colonization in the kidneys in the absence of bladder colonization is contrary to findings from intravesical inoculation of CBA/J mice with UPEC strain CFT073, where colonization is detected in both the bladders and kidneys. We tested whether UPEC entered the peritoneal cavity from the genital tract before dissemination to kidneys and systemic sites. UPEC was not detected by plate counts in peritoneal lavage fluid from these mice (data not shown). In summary, estrus and metestrus emerged as the stages of the estrous cycle that were most permissive to UPEC colonization in the murine urogenital tract. Since mice in estrus showed a more consistent colonization phenotype than mice in other stages of the estrous cycle, the rest of the experiments described in this report were performed during estrus.

### Temporal changes in UPEC colonization in mice during estrus.

**FIG 4:**
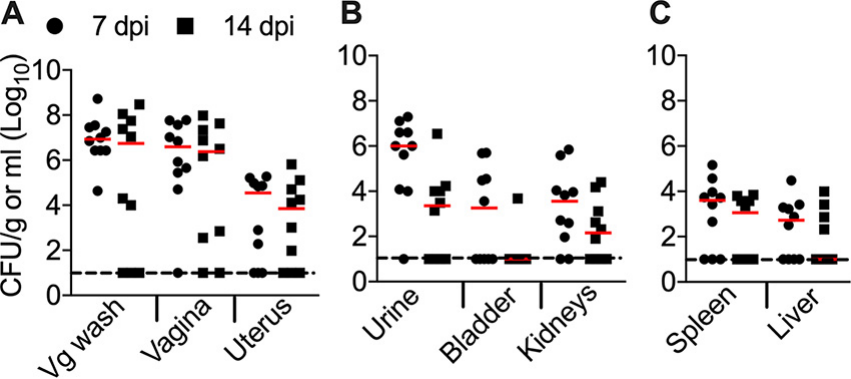
UPEC colonizes the urogenital tract of mice in estrus for at least 2 weeks. Female CBA/J mice (*n* = 10 mice/group) in estrus were inoculated with UPEC strain CFT073 in the vaginal tract. Urine and vaginal lavage fluid (Vg wash) were collected at day 7 or 14 postinoculation, prior to euthanasia. Bacterial loads in the samples were determined by viable counts and normalized to the volume/weight of the samples. Each symbol corresponds to results from a mouse, and red bars indicate medians. The dotted lines correspond to the limit of detection (10 CFU). dpi, day postinoculation.

To test whether the colonization observed at 1 day postinoculation was transient, temporal changes in bacterial colonization were determined 1 and 2 weeks after inoculation. Mice were inoculated during estrus, and the tissue bacterial burden was determined at later time points. Overall, trends in bacterial loads across the samples were conserved at the 1- and 2-week time points (Fig. 4A to C). There were no statistically significant differences in the bacterial burdens between the 1- and 2-week endpoints (Fig. 4A to C). We observed an uptick in the median bladder bacterial load at 1 week postinoculation (Fig. 4B), but this difference was not statistically significant. Bacterial loads at these later endpoints were also comparable to that at the 1-day endpoint (Fig. 3 and 4). Collectively, our data reveal persistent colonization by UPEC in the urogenital tract for up to 2 weeks after a single vaginal instillation during estrus.

### Comparison of UPEC colonization in mice inoculated by vaginal and transurethral routes.

**FIG 5:**
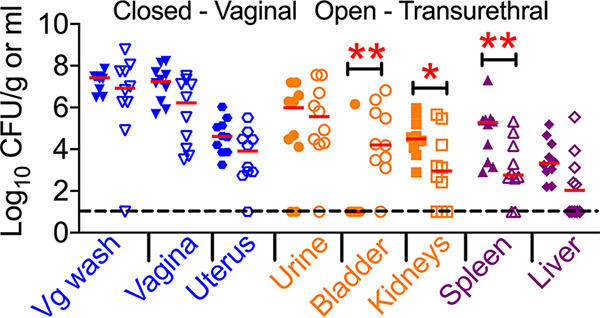
UPEC colonization after vaginal and transurethral inoculation. Female CBA/J mice (*n* = 10 mice/group) in estrus were inoculated with UPEC strain CFT073 in the vaginal tract (closed symbols) or transurethrally in the bladder (open symbols). Urine and vaginal lavage fluid (Vg wash) were collected at 1 day postinoculation, and mice were euthanized. Bacterial loads in the samples were determined by viable counts and normalized to the volume/weight of the samples. Each symbol corresponds to results from a mouse, and red bars indicate medians. The dotted line corresponds to the limit of detection (10 CFU). Blue, genital tract; orange, urinary tract; purple, systemic sites. *, *P < *0.05; **, *P < *0.01 (by a Mann-Whitney test).

To further explore the impact of estrus on the colonization of the urogenital tract, we directly compared the outcomes of vaginal and transurethral routes of inoculation. Mice (*n* = 10 mice/group) in estrus were inoculated either in the vagina or in the urinary bladder with a transurethral catheter, as described in Materials and Methods. As expected, intravesical instillation of UPEC led to a higher bacterial burden in the bladder than vaginal inoculation (Fig. 5). However, mice inoculated in the vaginal tract carried higher bacterial burdens in the kidneys than those inoculated transurethrally (Fig. 5). Both routes of inoculation resulted in comparable colonization of the genital tract and urine with UPEC strain CFT073 (Fig. 5). Increased systemic dissemination from the urogenital tract was observed after vaginal instillation, and this difference was statistically significant in the spleen (Fig. 5). Both vaginal and intravesical instillation of UPEC resulted in genital tract colonization (Fig. 5). In the urinary tract, vaginal inoculation led to renal colonization, whereas intravesical inoculation led to colonization of both the bladder and kidneys.

### Tissue changes in the urogenital tract after UPEC inoculation in the vagina.

We next assessed if instillation of UPEC in the vagina resulted in an active infection, defined as a combination of pathogen colonization, inflammation, and damage in the tissues. Levels of key cytokines (interleukin-1β [IL-1β], IL-6, and tumor necrosis factor alpha [TNF-α]) and myeloperoxidase (MPO) (an indicator for neutrophil abundance) in the urine, bladder, kidneys, vagina, and uterus were determined by an enzyme-linked immunosorbent assay (ELISA) (Fig. S1). Levels of these analytes in UPEC-instilled mice were compared to those in phosphate-buffered saline (PBS)-instilled or unmanipulated naive mice at the 1-day endpoint, as reported by us and others (27–29). There were no significant differences in the levels of these analytes as determined by a semiquantitative ELISA between groups at this early time point (Fig. S2).

**FIG 6:**
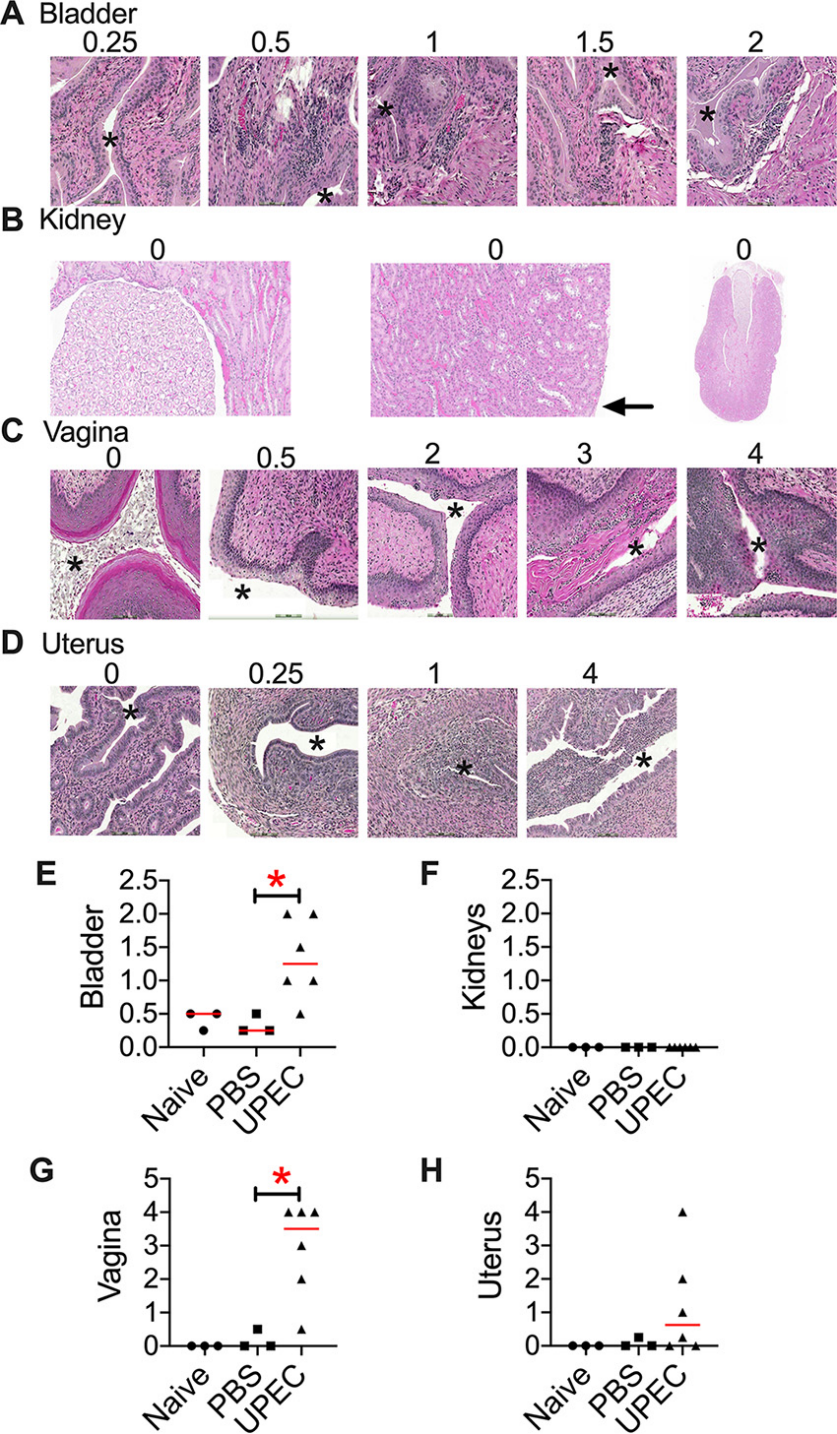
Histopathological changes induced by UPEC in the urogenital tract. Female CBA/J mice (*n* = 3 to 6/group) in estrus were inoculated with UPEC strain CFT073 in the vaginal tract. Organs were collected after euthanasia at 1 day postinoculation. H&E-stained sections were evaluated by light microscopy. (A to D) Representative images for each tissue damage score (magnification, ×200). A low-magnification (×20) image of an entire kidney is included in panel B. Lumina in the bladder, vagina, and uterus are indicated with asterisks. The black arrow in panel B points to the renal serosa. (E to H) Each symbol corresponds to results from a mouse, and red bars indicate medians. *, *P < *0.05 (by a Mann-Whitney test).

Next, we determined if UPEC colonization in the urogenital tract led to tissue damage by evaluating tissue sections. A board-certified veterinary anatomic pathologist who was blind to the study groups assigned inflammation and tissue damage scores as described in Materials and Methods. UPEC-instilled mice had a higher degree of inflammatory changes in the urinary bladder than the controls (Fig. 6A and E). Bladders from UPEC-infected mice revealed mild-to-moderate cystitis, with higher tissue damage/inflammation scores than the control groups (Fig. 6A). We did not observe signs of inflammation and tissue damage in the kidneys despite their elevated bacterial loads, suggesting bacterial colonization that did not progress to an active infection (Fig. 6B and F). UPEC caused a significant increase in pathological changes observed in the vagina (Fig. 6C and G), compared to the control groups. Lesions in the vagina of UPEC-infected mice were consistent with those of acute purulent vaginitis (Fig. 6C). Although UPEC induced pathological changes in the uterus of some infected mice (Fig. 6D), this change was not statistically significant (Fig. 6H).

### UPEC strain-dependent pattern of urogenital colonization following vaginal instillation.

**FIG 7:**
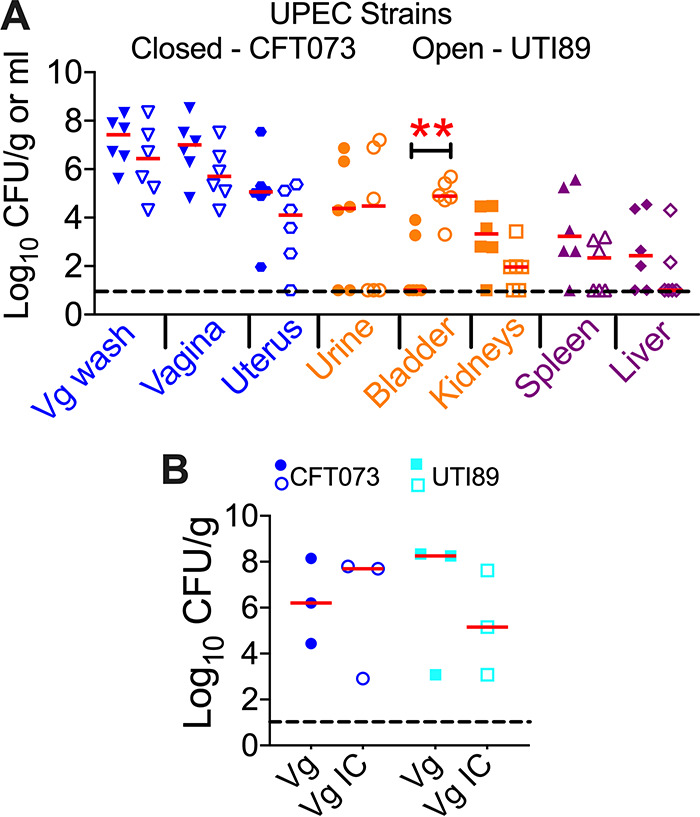
UPEC strains CFT073 and UTI89 colonize the urogenital tract of mice in estrus. (A) Female CBA/J mice (*n* = 6 mice/group) were inoculated with UPEC strain CFT073 (closed symbols) and UTI89 (open symbols) in the vaginal tract. Urine and vaginal lavage fluid (Vg wash) were collected at 1 day postinoculation, prior to euthanasia. Bacterial loads in the samples were determined by viable counts and normalized to the volume/weight of the samples. Blue, genital tract; orange, urinary tract; purple, systemic sites. (B) Female CBA/J mice (*n* = 3 mice/group) were inoculated with UPEC strain CFT073 (blue circles) and UTI89 (cyan squares) in the vaginal tract. Mice were euthanized at 1 day postinoculation, and vaginas (Vg) were collected. The total bacterial vaginal bacterial loads are depicted as solid symbols. Gentamicin protection assays were performed to determine vaginal intracellular UPEC loads (open symbols). Each symbol corresponds to results from a mouse, and red bars indicate medians. The dotted lines correspond to the limit of detection (10 CFU). *, *P < *0.05; **, *P < *0.01 (by a Mann-Whitney test). Vg IC, vagina intracellular.

Since UPEC strains are genetically heterogeneous, we evaluated the outcomes of vaginal instillation of two prototypical strains of UPEC (CFT073 and UTI89). Mice (*n* = 6/group in estrus) in both groups showed high levels of colonization in the urogenital tract, urine, and vaginal lavage fluid, except in the urinary bladder (Fig. 7A). Bladder colonization was significantly higher in mice inoculated with UPEC strain UTI89 than in mice inoculated with CFT073 (Fig. 7A). However, in most mice inoculated with UPEC strain CFT073 (4 out of 6), bacterial loads were below the limit of detection in the bladder (Fig. 7A) and were aligned with our above-described findings (Fig. 3 and 5).

### UPEC invades vaginal tissue following colonization.

UPEC has been demonstrated to invade and establish an intracellular niche in the vagina (24). Here, we tested whether vaginal colonization in mice during estrus leads to invasion and intracellular persistence at 1 day postinoculation. Vaginal tissues from mice in estrus instilled with UPEC strains CFT073 and UTI89 were exposed to gentamicin to kill extracellular bacteria on the mucosal surface. Plate counts were performed to determine total and intracellular bacterial loads in the vagina. Our results reveal that vaginal instillation of clinical strains of UPEC results in invasion and intracellular colonization in the murine vagina (Fig. 7B). Importantly, both prototypical strains of UPEC (CFT073 and UTI89) invaded and persisted within vaginal tissue (Fig. 7B).

### Urogenital UPEC colonization is not due to contamination from bedding.

**FIG 8:**
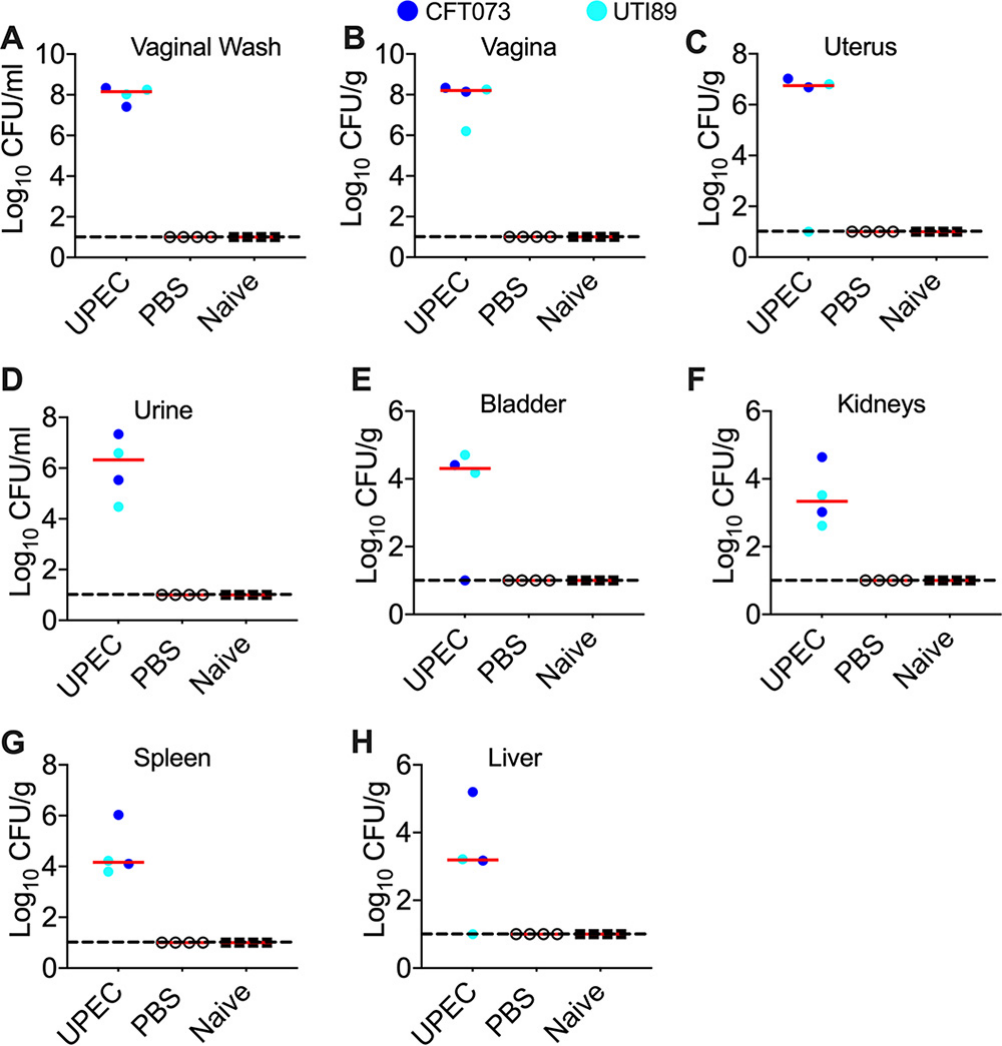
Contaminated bedding is not a source of urogenital UPEC colonization. Female CBA/J mice in estrus were inoculated with UPEC strains CFT073 (*n* = 2) (blue circles) and UTI89 (*n* = 2) (cyan circles) in the vaginal tract. These mice were cohoused with a PBS-instilled mouse and a naive control mouse such that each cage had two UPEC-instilled and two control mice. Mice were euthanized after the collection of urine and vaginal lavage fluid at 1 day postinoculation. Organs were collected to determine bacterial loads (A-H). Each symbol corresponds to results from a mouse, and red bars indicate medians. The dotted lines correspond to the limit of detection (10 CFU).

Next, we tested whether vaginal inoculation leads to sustained UPEC colonization or is a result of exposure to contaminated bedding. To address this question, mice in estrus were inoculated with UPEC strain CFT073 or UTI89 (one UPEC strain/cage) and cohoused with PBS-instilled and naive mice. Animals were euthanized at 1 day postinoculation, and the bacterial load was determined. Our results revealed that mice that were instilled with clinical strains of UPEC (CFT073 and UTI89) were colonized by UPEC in the genital tract (Fig. 8A to C), urinary tract (Fig. 8D to F), and systemic sites (Fig. 8G and H). However, PBS or naive controls remained UPEC free at all tested sites, indicating that mice are stably colonized by UPEC following vaginal instillation (Fig. 8A to H), ruling out ongoing contamination from bedding as a source of UPEC in the urogenital tract.

## DISCUSSION

UTI is among the top bacterial infections encountered by people throughout the world (7, 30). Mouse models of UTI have been used extensively to elucidate host and pathogen factors that are involved in the pathogenesis of UTI and to test the efficacy of therapeutics and prophylactics (15, 18, 19). These models were designed to investigate UPEC colonization, ensuing infection and inflammation in the urinary bladder and renal pelvis, and systemic dissemination from the urinary tract. Existing models either do not involve vaginal inoculation or utilize estrogenized mice (24, 31) to probe UPEC vaginal colonization and UTI. The administration of exogenous estrogen has been demonstrated to increase the susceptibility of the female genital tract and kidneys to bacterial infections (32–34). Adult, reproductive-age women represent a preponderance of patients affected by UTI (6, 30). Here, we sought to determine the effect of the stage of the estrous cycle on UPEC colonization in the urogenital tract of adult female mice that were not administered exogenous estrogen. Our approach more closely emulates the physiological changes observed in the adult female genital tract.

By comparing various stages of the estrous cycle, our study demonstrates the impact of endogenous changes in the levels of various hormones on UPEC colonization in the murine urogenital tract. The results reported here demonstrate that estrus and metestrus are the most permissive phases of the estrous cycle for colonization of the genital tract, urine, and kidneys and systemic dissemination. Importantly, single inoculation in the vaginal tract leads to colonization for at least 14 days in the urogenital tract and systemic sites. It is well documented that estrus is preceded by elevated levels of estrogen and decreased levels of progesterone, compared to other stages of the estrous cycle in mice (35, 36). Our findings that mice in estrus are more susceptible to UPEC colonization are bolstered by results from estrogenized mice that also demonstrate increased UPEC colonization, compared to controls (24, 31, 34). A protective role for estrogen against bacterial colonization was detected in ovariectomized mice, which were more highly colonized by UPEC during UTI than the controls (37). Our results are congruent with the established clinical observations that postmenopausal women are at a higher risk for developing UTI (7, 23) and that estrogen has therapeutic value in decreasing UTI incidence and UPEC colonization in this cohort (38–40). Additionally, signaling through estrogen receptors modulates UPEC invasion in a human urothelial cell line *in vitro* (41). Collectively, these findings reveal an important role for decreased estrogen levels as a determinant of bacterial colonization in the urogenital tract.

Nucleated and cornified squamous epithelial cells are shed from the vaginal mucosa during proestrus and estrus, respectively. The presence of these cell types is routinely utilized to establish proestrus and estrus phases by vaginal cytology, including in this study (42, 43). Exfoliation of superficial epithelial cells in the urinary bladder is a well-characterized host response to UTI (44–46). We hypothesized that exfoliated vaginal epithelial cells would act as decoys to protect against epithelial colonization during proestrus and estrus. However, we observed poor colonization during proestrus and robust colonization during estrus in the vaginal tract, indicating that exfoliated nucleated and cornified epithelial cells exert disparate effects on UPEC colonization.

Stages of the estrous cycle are associated with profound changes in tissue structure, luminal contents, immune cell infiltration, and cytokine levels in the reproductive tract. Histopathological evaluation indicates the induction of inflammation in the vagina and urinary bladder in UPEC-instilled mice (Fig. 6). However, semiquantitative measurements of cytokine and myeloperoxidase levels did not reveal statistically significant differences (see Fig. S2 in the supplemental material). Considering known estrous cycle-dependent changes in cytokine expression in the female genital tract (47), our results suggest that the baseline changes during estrus mask the differences in cytokine and myeloperoxidase levels among mice in the naive, PBS-instilled, and UPEC-instilled groups. The divergence of the results from cytokine measurements and histopathology evaluation is likely the result of capturing UPEC-induced structural changes in tissues in the latter analyses. The extent to which UPEC colonization affects changes in cytokine expression in the urogenital tract during various stages of the estrous cycle remains to be determined and is a logical extension of this study. Additionally, quantitative ELISAs and flow cytometry should be included in follow-up studies to develop a comprehensive portrait of immune cell and effector changes elicited by UPEC during various stages of the estrous cycle.

UPEC is a collection of genetically heterogeneous bacterial pathogens that are usually derived from E. coli belonging to phylogroups B2 and D (30, 48). A key finding from our study is that genetic differences in UPEC strains determine the potential to colonize the urinary bladder following vaginal inoculation during estrus. UPEC strain CFT073 does not colonize the urinary bladder when inoculated in the vagina, as opposed to strain UTI89, which demonstrates superior bladder colonization. This difference could be attributed to the original sites of infection caused by these prototypical UPEC strains. Strain UTI89 was isolated from a patient with cystitis, whereas strain CFT073 was isolated from a patient with pyelonephritis and bacteremia (49, 50). UPEC strain-specific effects should be considered during the application of the model described in this report.

The vagina has been proposed as an intermediary niche for UPEC during its transmission from the gut to the urinary tract. UPEC strains are known to establish intracellular reservoirs in the bladder epithelium, and these reservoirs have been implicated in the development of recurrent UTI (46, 51, 52). Recently, Brannon et al. demonstrated the presence of intracellular UPEC in vaginal epithelial cells from patients suffering from recurrent UTI, highlighting the importance of the vaginal epithelium as a niche in the pathogenesis of UTI (24). They also established a mechanism by which UPEC invades and forms intracellular reservoirs in human vaginal epithelial cells (24). Our study demonstrates that UPEC stably colonizes cycling, adult female mice that are not treated with exogenous estrogen. Here, we demonstrate that UPEC strains CFT073 and UTI89 invade vaginal tissue by using gentamicin protection assays in mice during estrus. Further studies are required to evaluate the cellular localization of UPEC in this mouse model. There is the potential for reinoculation of UPEC into the urogenital tract from contaminated bedding that could be mistaken for stable colonization. Cohousing of UPEC-instilled and control mice demonstrates that UPEC colonizes mice in estrus and is not reintroduced from the bedding since the controls remained UPEC free (Fig. 8). The model reported in this study could be applied to interrogate mechanistic questions on the role of the vagina as a reservoir for pathogens affecting the female urogenital tract under physiological conditions.

Despite the comparable anatomic structures of human and mouse vaginas, notable differences have also been described. The vaginal microbiomes are profoundly different between these hosts, with humans harboring a *Lactobacillus-*predominant microbial community that leads to an acidic pH from puberty through menopause (53). The mouse vaginal microbiome is typically rich in Streptococcus, and the pH of vaginal secretions is near neutral (53, 54). There is also an increasing interest in understanding the role of the vaginal microbiome in UTI outcomes and developing vaginal microbiome-based approaches to deter UPEC colonization (55–57). Therefore, the results from this report and others that utilize a vaginal route of inoculation (31) must be interpreted while being cognizant of the species-specific differences. An avenue to refine the model presented here would be to develop and utilize mice with a humanized vaginal microbiome.

While establishing a simple and physiological murine model of female urogenital tract colonization, our findings also raise important questions for further investigation. Here, we utilized adult female mice to test the effect of the stage of the estrous cycle and associated changes in hormone levels on UPEC colonization. Outcomes of vaginal instillation of UPEC in prepubertal and postmenopausal mice should be evaluated to understand the role of reproductive status in determining susceptibility to urogenital infections. We used CBA/J mice since they develop UTI with a more robust renal involvement. The effect of the genetic background of a mouse strain on the outcomes of urogenital infection is another question that needs to be tested in future studies. In summary, our findings demonstrate that mice in estrus are highly susceptible to UPEC colonization of the genital tract and kidneys along with systemic dissemination following instillation in the vagina.

## MATERIALS AND METHODS

### Mice.

Experiments were conducted in accordance with the Animal Welfare Act, and all protocols were approved by the Institutional Animal Care and Use Committee of Texas A&M University (IACUC-2018-0362). CBA/J female mice (6 to 8 weeks old; JAX Laboratories) were housed under specific-pathogen-free conditions in an AAALAC-accredited biosafety level 2 (BSL2) animal facility at Texas A&M University. Mice were assessed for pathogen status, by surveillance testing using dirty bedding sentinels and exhaust air dust, for the following agents: lymphocytic choriomeningitis virus, mouse adenovirus, Mycoplasma pulmonis, Theiler murine encephalomyelitis virus, pneumonia virus of mice, reovirus, Sendai virus, mouse hepatitis virus, minute virus of mice, mouse parvovirus, mouse rotavirus, ectromelia virus, polyomavirus, pinworms, and fur mites. All mice were negative for the above-mentioned pathogens for the duration of the study.

### Vaginal cytology.

Mice were acclimated to our housing facility for at least 2 days before the collection of samples. Mice were restrained by scruffing and immobilizing the base of the tail for the collection of vaginal lavage fluid with 50 μL of sterile deionized water dispensed from a micropipette. Smears of vaginal lavage fluid were prepared, stained with DiffQuick (Fisher Scientific), and evaluated by light microscopy. Stages of the estrous cycle were determined based on the presence and abundance of nucleated epithelial cells, cornified epithelial cells, and/or leukocytes, as described previously (42, 43). Vaginal cytology was performed for 4 consecutive days, followed by a rest phase of a week, and repeated prior to inoculation of UPEC. A rest phase was included to avoid accidental induction of anestrus.

### Bacterial strains and growth conditions.

Clinical UPEC strains CFT073 and UTI89 (49, 50) and CFT073 mutants with the type 1 pilus promoter locked in the on or off orientation (58) were cultured in LB broth or agar (tryptone at 10 g/L, yeast extract at 5 g/L, NaCl at 5 g/L, and agar at 15 g/L). Cultures were incubated at 37°C and aerated by shaking at 200 rpm. UPEC strains were cultured to stationary phase in LB broth, washed, and resuspended in PBS prior to inoculation in mice.

### Hemagglutination assays.

Hemagglutination titers were determined using guinea pig and human erythrocytes to assess the presence of type 1 and P pili, respectively (25, 26). The inoculum, prepared as described above, was serially diluted 2-fold in PBS and mixed with an equal volume of a 3% (vol/vol) suspension of erythrocytes (Innovative Research). Strains CFT073 fim-ON and fim-OFF were used as positive and negative controls for type 1 pilus-dependent hemagglutination. The mannose dependence of hemagglutination was validated with α-methyl d-mannopyranoside (10 mg/mL; Sigma). Plates were incubated at room temperature for 30 min. The reciprocal of the fold dilution of bacteria in the last well with agglutination was recorded as the hemagglutination titer. The experiment was repeated independently, and results were analyzed by a *t* test.

### Vaginal instillation of UPEC.

Mice (*n* = 10/group/time point) were instilled with approximately 2 × 10^8^ CFU/mouse of UPEC strain CFT073 or UTI89 in 50 μL of PBS in the vaginal tract. Urine and vaginal lavage fluid were collected prior to euthanasia (1, 7, or 14 days after inoculation). After euthanasia, blood, vagina, uterus, bladder, kidneys, spleen, and liver were collected aseptically, homogenized in PBS, and cultured on LB agar. After 1 day of incubation, viable bacterial counts were determined to assess genital, urinary tract, and systemic colonization.

### Cohousing after vaginal UPEC instillation.

To rule out contamination by UPEC from bedding, we performed cohousing experiments with UPEC-instilled, PBS-instilled, and naive mice. Mice in all groups were in estrus during UPEC/PBS instillation. Mice were vaginally inoculated with UPEC strain CFT073 or UTI89 and cohoused with control mice in a cage (one UPEC strain/cage) with autoclaved bedding for 1 day. Samples were collected and processed at 1 day postinoculation, as described above.

### Gentamicin protection assay to determine intracellular UPEC loads.

In an independent experiment, vaginas from mice in estrus that were infected with UPEC strain CFT073 or UTI89 intravaginally were collected at 1 day postinoculation and bisected along the longitudinal axis. One half was homogenized to determine the total (extracellular and intracellular) UPEC load. A gentamicin protection assay was performed as reported previously by Brannon et al. (24). Briefly, the other half of the vagina was rinsed in PBS, treated with gentamicin (100 μg/mL) for 2 h, and extensively rinsed in PBS prior to homogenization to determine plate counts.

### Intravesical instillation of UPEC.

Mice were anesthetized with tribromoethanol, and the bladder was catheterized transurethrally. UPEC, comparable to the inoculum instilled in the vagina, was instilled in the bladder, as we and others have described previously (15, 27, 59). Sample collection and tissue processing were performed essentially as mentioned above for the vaginal UPEC instillation experiments.

### Histopathology.

The vagina, uterus, bladder, and kidneys (entire organs) from naive, PBS control, and UPEC-inoculated mice were fixed in formalin, embedded in paraffin, and sectioned. Hematoxylin and eosin (H&E)-stained sections were evaluated by a board-certified veterinary anatomic pathologist who was blind to study groups. Tissue damage scores were assigned based on the degree of inflammation, immune cell infiltration, integrity of the mucosa, the presence and nature of luminal contents, exfoliation of epithelial cells, tissue edema, the presence and extent of inflammatory exudate, microabscess formation, the presence or absence of bacteria, and the presence or absence of tissue injury. Tissue scores were assessed on a scale of 0 (healthy) through 4 (extensive inflammation and damage).

### ELISA.

Urine and homogenates of the vagina, uterus, bladder, and kidneys were used to determine the abundances of myeloperoxidase (MPO), IL-1β, IL-6, and TNF-α, as we have recently described (28, 59). Urine samples were normalized based on the creatinine content, and tissue samples were normalized to the weight of organs. Optical density values were used for semiquantitative comparison of between-group changes in the analyte levels.

### Statistical analysis.

Experiments were repeated at least twice independently. Results were analyzed in Prism 7 (GraphPad) by a *t* test, a Mann-Whitney U test, or analysis of variance (ANOVA). A *P* value of <0.05 was considered a statistically significant difference.
