# Supplementary material for: Vaginal Inoculation of Uropathogenic Escherichia coli during Estrus Leads to Genital and Renal Colonization
Source: Infect Immun. 2022 Mar 31;90(4):e00532-21. doi: 10.1128/iai.00532-21 (PMC9022555; doi:10.1128/iai.00532-21)
Supplement: Supplemental file 1 — Fig. S1 and S2. Download iai.00532-21-s0001.pdf, PDF file, 0.2 MB [file iai.00532-21-s0001.pdf]

SUPPLEMENTARY MATERIAL

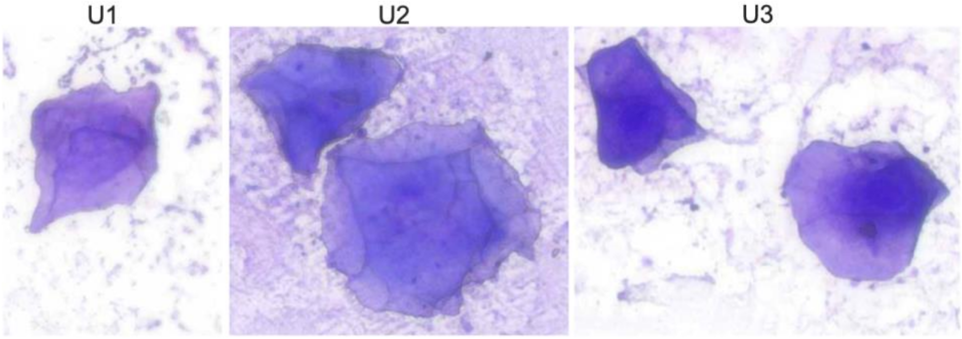

Fig. S1. Urine cytology analysis. Urine samples were collected from mice in estrus instilled with UPEC strain CFT073 in the vaginal tract. Cells were stained with DiffQuick and evaluated by light microscopy. Representative images from urine samples of three mice are depicted here (400X magnification).

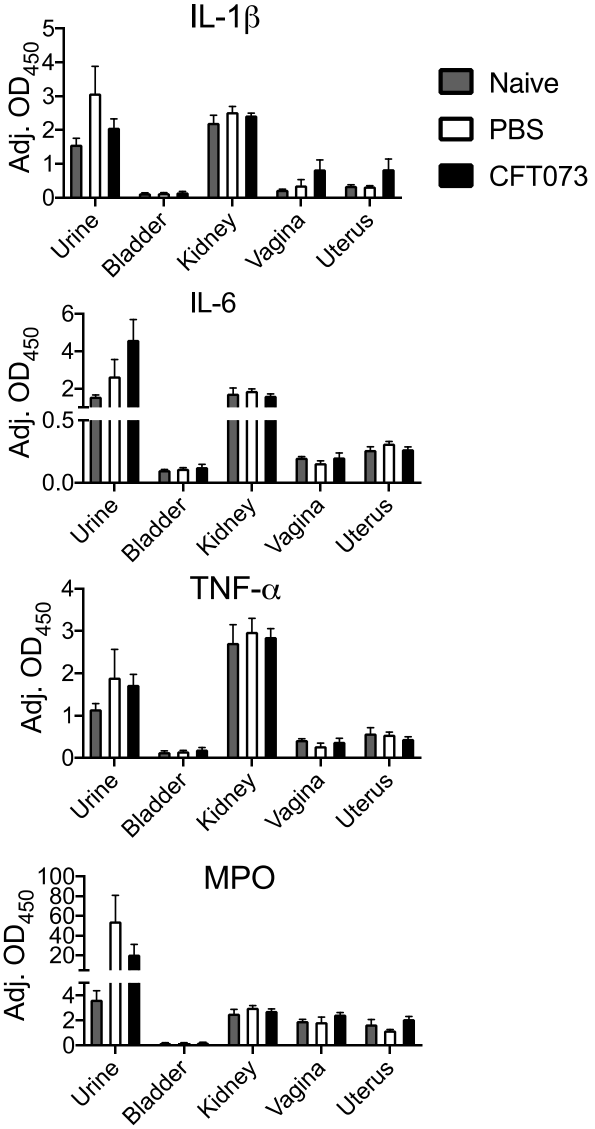

Fig. S2. Cytokine response and neutrophil mobilization to the urogenital tract after UPEC inoculation. Female CBA/J mice (N=3-6 mice/group) were inoculated with UPEC strain CFT073 in the vaginal tract. Naïve, and PBS-inoculated mice were used as controls. Urine was collected one day post-inoculation, prior to euthanasia. Organs were homogenized and clarified homogenates were utilized for ELISA to detect IL-1β, IL-6, TNF-α, and MPO. OD values were normalized to organ weight or volume of urine. Mean and SEM are depicted here.
